# Editorial: Liquid biopsy in non-small cell lung cancer for diagnosis, treatment selection and monitoring

**DOI:** 10.3389/fonc.2026.1929637

**Published:** 2026-07-23

**Authors:** Areti Strati, Benoit Roch, Ammar Sukari

**Affiliations:** 1 Department of Biological Chemistry, Medical School, National and Kapodistrian University of Athens, Athens, Greece; 2 Thoracic Oncology Unit, Department of Respiratory Diseases, University of Montpellier, Montpellier University Hospital, Montpellier, France; 3 Department of Oncology, Karmanos Cancer Institute, Wayne State University, Detroit, MI, United States

**Keywords:** Liquid Biopsy, ctDNA, Lung Cancer, NSCLC, AI

Lung cancer is the third most common malignancy in the United States and remains the leading cause of cancer-related mortality worldwide. Despite ongoing advances in diagnosis and therapy, survival rates for patients with lung cancer remain unsatisfactory (1). In recent years, interest in liquid biopsy (LB) has grown substantially, particularly following the approval of LB testing in lung cancer. Liquid biopsy has the potential to transform clinical management by enabling minimally invasive, real-time molecular assessment (2). The studies included in this Research Topic illustrate how rapidly the field is evolving—from case-based therapeutic insights to quantitative imaging approaches, metabolomic signatures, and dynamic ctDNA monitoring.

Epidermal growth factor receptor (EGFR) tyrosine kinase inhibitors (TKIs), such as erlotinib, represent one of the most important classes of targeted therapies for non–small cell lung cancer (NSCLC), particularly in tumors harboring activating EGFR mutations (3). However, evidence supporting their benefit in leptomeningeal carcinomatosis remains limited (4). Neira et al. describe an exceptional case involving a 42-year-old patient with EGFR-mutant NSCLC who developed secondary leptomeningeal carcinomatosis, a condition typically associated with an extremely poor prognosis. The patient received an intensive multimodal regimen consisting of intrathecal chemotherapy, craniospinal radiotherapy, and prolonged erlotinib therapy at 150 mg/day for five years. Remarkably, the patient remained disease-free and no EGFR mutation was detected via liquid biopsy, suggesting that ctDNA negativity may serve as a surrogate marker of durable remission (4). When ctDNA levels become undetectable during or after therapy, this often reflects a profound reduction in tumor burden and correlates with improved clinical outcomes, including longer progression-free and overall survival (5).

Building on this observation, two studies in this Research Topic further examine the clinical significance of ctDNA clearance, highlighting its emergence as a robust biomarker of treatment response (6, 7). The meta-analysis by Li et al. demonstrates that ctDNA clearance during EGFR-TKI therapy is strongly associated with improved outcomes in unresectable EGFR-mutant NSCLC. As the authors note, “ctDNA dynamics may serve as a promising early prognostic biomarker,” although assay standardization remains essential (7). Complementary findings from an aggregate analysis of eight clinical trials reinforce that patient-level ctDNA kinetics are closely linked to clinical benefit (8). In locally advanced NSCLC, ctDNA MRD negativity after two cycles of neoadjuvant chemoimmunotherapy predicted benefit from additional treatment, with the highest pCR rate (75%) observed in patients who maintained MRD clearance. These results support a future in which ctDNA MRD guides the intensity and duration of neoadjuvant therapy, enabling adaptive treatment strategies and allowing pathological response to be anticipated before surger (6). Together, these studies underscore ctDNA as a dynamic, real-time biomarker capable of informing prognosis, therapeutic sequencing, and individualized treatment planning.

Primary tumor location is a recognized prognostic factor and has been shown to be highly significant in clinical decision-making in cancer (9). Although substantial advances have been made in preoperative and intraoperative localization techniques for pulmonary nodules, important limitations remain despite this progress (10). Yang et al. explored an innovative aspect of early-stage NSCLC biology by investigating whether inflammatory lipid indices and serum tumor markers could predict the anatomical location of pulmonary lesions. Their multivariable analysis demonstrated that these biomarkers were independent predictors of lower lobe tumor location in patients with early-stage NSCLC (11). Integrating these biomarkers into artificial intelligence (AI)-based predictive models may further enhance their clinical utility.

In parallel, Ruan et al. developed and internally validated an AI-assisted clinicoradiological quantitative imaging nomogram to improve the preoperative assessment of malignancy risk in solid and part-solid pulmonary nodules ≤3 cm in diameter (12). The model integrates automatically extracted quantitative CT imaging features with clinical variables and inflammatory biomarkers. To facilitate clinical implementation, the authors also created an accessible online risk calculator. However, they emphasized that “these risk estimates should be interpreted within a malignancy-enriched preoperative surgical setting,” reflecting the characteristics of the study population (12). The integration of multidimensional clinical, imaging, and molecular data through advanced bioinformatics and AI-based approaches enables the development of robust predictive algorithms and clinical decision-support systems, thereby advancing precision medicine and personalized therapeutic strategies (2).

A machine learning-driven predictive model based on pathway-oriented plasma metabolomic biomarkers for lung cancer detection was reported by Himdiat et al. Using a large plasma metabolomics dataset, the authors identified 41 key predictive features through an ensemble feature-selection framework. Notably, reducing the number of variables preserved biological interpretability while minimizing the risk of overfitting, with glutaminolysis and tryptophan metabolism emerging as the most informative metabolic pathways (13). Consistent with these findings, metabolomic alterations across multiple biological fluids have been observed when comparing patients with lung cancer and healthy controls (14). Furthermore, a classifier based on only five metabolites accurately distinguished both lung cancer stages and histological subtypes, underscoring the potential for translating metabolomics-based diagnostics into point-of-care devices for early lung cancer detection, classification, and prognostication (15).

Taken together, the contributions in this Research Topic highlight the accelerating convergence of liquid biopsy technologies, quantitative imaging, metabolomics, and machine-learning approaches in reshaping lung cancer diagnostics and therapeutic decision-making. Across diverse methodologies and clinical settings, a unifying theme emerges: minimally invasive, data-rich biomarkers are beginning to capture tumor biology with a level of precision that complements—and in some cases surpasses—traditional modalities. As these innovations progress toward broader validation and clinical integration, they hold the promise of enabling earlier detection, more accurate risk stratification, and truly adaptive treatment strategies. Continued investment in prospective studies, assay harmonization, and multi-omic integration will be essential to realizing a future in which lung cancer care is guided by dynamic, individualized, and biologically informed tools (2).
